# An Improved Healthcare Accessibility Measure Considering the Temporal Dimension and Population Demand of Different Ages

**DOI:** 10.3390/ijerph15112421

**Published:** 2018-10-31

**Authors:** Lan Ma, Nianxue Luo, Taili Wan, Chunchun Hu, Mingjun Peng

**Affiliations:** 1 School of Geodesy and Geomatics, Wuhan University, Wuhan 430079, China; lanma_sgg@whu.edu.cn (L.M.); wantaili@whu.edu.cn (T.W.); chchhu@sgg.whu.edu.cn (C.H.); 2 Wuhan Land Resources and Planning Bureau, Wuhan 430014, China; Pmj@wpl.gov.cn

**Keywords:** healthcare accessibility, population demand, geographic impedance, the elderly, urban planning, 3SFCA, real-time traffic, crowdedness

## Abstract

Healthcare accessibility has become an issue of social equity. An accurate estimation of existing healthcare accessibility is vital to plan and allocate health resources. Healthcare capacity, population demand, and geographic impedance are three essential factors to measure spatial accessibility. Additionally, geographic impedance is usually represented with a function of travel time. In this paper, the three-step floating catchment area (3SFCA) method is improved from the perspectives of the temporal dimension and population demand. Specifically, the travel time from the population location to the service site is precisely calculated by introducing real-time traffic conditions instead of utilizing empirical speed in previous studies. Additionally, with the utilization of real-time traffic, a dynamic result of healthcare accessibility is derived during different time periods. In addition, since the medical needs of the elderly are higher than that of the young, a demand weight index of demand is introduced to adjust the population demand. A case study of healthcare accessibility in Wuhan shows that the proposed method is effective to measure healthcare accessibility during different time periods. The spatial accessibility disparities of communities and crowdedness of hospitals are identified as an important reference for the balance between the supply and demand of medical resources.

## 1. Introduction

Healthcare accessibility is the relative convenience of achieving healthcare services at a certain location [1]. By studying the disparities of spatial distribution between medical resources and inhabitants, areas lacking medical resources are revealed. Thus, an accurate estimation of existing healthcare accessibility is of great significance for government departments to make scientific decisions on urban planning so as to guarantee the proper allocation of medical resources.

The concept of access to healthcare has evolved during the last decades. Penchansky and Thomas [2] conceptualized access into five specific dimensions to describe the fit between the patient and the healthcare system. Additionally, these dimensions are availability, accessibility, accommodation, affordability, and acceptability. In recent studies, this concept was introduced by describing broad dimensions. As described by Levesque et al. [3], access encompassed five dimensions: approachability, acceptability, availability and accommodation, affordability, and appropriateness. Saurman [4] argued that awareness should be another dimension of access and modified Penchansky and Thomas’s Theory of access. Access to healthcare is decided by the spatial accessibility of healthcare services, which is a primary deciding factor of healthcare utilization [5].

As to measure and evaluate healthcare accessibility, three factors are essential: healthcare capacity, population demand, and geographic impedance [6,7,8]. Healthcare capacity is the supply of healthcare services. Additionally, it can be represented by using the amount of specific facilities, physicians, or sickbeds. Population demand means the number of people who may need the services. Geographic impedance indicates to what extent the ‘distance’ between service location and population demand will affect accessibility. The ‘distance’ is usually characterized by travel time.

For patients, especially with acute diseases, an accurate estimation of travel time from the population location to hospitals is of crucial importance. In previous research, the classic method of simulating travel time to assign each road an empirical speed and then conduct a road network analysis [9,10,11]. However, with the development of urbanization, tidal transportation is a common phenomenon nowadays and road congestion varies during different time periods (e.g., at rush hours or at non-peak periods). Without considering traffic conditions, the theoretical travel time given by the classic approach is usually inaccurate. Healthcare accessibility is a static result by given travel time in previous studies. However, when considering traffic conditions, accessibility is dynamic. In addition, the elderly are mostly valetudinarian and need more frequent communications with healthcare providers [12]. Given the same population, a community with a higher proportion of the elderly will need more medical resources than that with a lower proportion. However, existing studies rarely consider the impact of different ages on medical needs. Additionally, as a result, the population demand is underestimated, especially in areas where there are more old people.

With the popularization of low-cost GPS devices and the development of Location Based Services (LBS), commercial map companies such as Google, Baidu, and AutoNavi, can obtain the location of floating vehicles or public users. With the help of big data mining technology, real-time traffic conditions are derived and provided to the public for route planning. Nowadays, people prefer to select a route with the help of real-time traffic conditions to reduce travel time, e.g., searching for medical treatment.

The paper improves the three-step floating catchment area (3SFCA) method, a recent method to measure healthcare accessibility. Specifically, the travel time is precisely calculated by introducing real-time traffic conditions from AutoNavi. Additionally, with the utilization of real-time traffic, dynamic results of healthcare accessibility are derived during different time periods. Besides, the population demand is adjusted by a demand weight index that characterizes the difference in medical needs between the elderly and the young. This improved method is illustrated and validated by a case study of healthcare accessibility in Wuhan. Results show that this study is of great significance in guiding how to better plan urban medical facilities.

## 2. A Methodological Review

Numerous measures have been utilized to estimate spatial accessibility, including the regional availability model [13], the gravity model [14], and the two-step floating catchment area (2SFCA) [15] method. The regional availability method is simply the ratio of supply (the capacity of service site) and demand (population) within a given area (e.g., administrative boundary). However, this method has been criticized because of its unreasonable assumptions: people do not go beyond the given area to seek medical services and all individuals within the given area have equal access regardless of the distance decay. The gravity model assumes that one’s spatial access to medical services decreases with the increase of its distance to nearby medical sites in a gravitational way [14], which is theoretically sounder. However, it requires more computation and the result is not intuitive to interpret [16]. The 2SFCA is a special case of the gravity model but intuitive to interpret.

### 2.1. The Basic Two-Step Floating Catchment Area Method

Luo and Wang [15] developed the 2SFCA method and this method is conducted in two steps:

Step 1: Generate a zone (or catchment) with a threshold travel cost (*d*_0_) for each service site *j* and search all population locations within the catchment. The supply-to-demand ratio *R_j_* for each service site *j* is calculated according to the site’s capacity and population demand with the following equation:

(1) $$R_{j}=\frac{S_{j}}{\sum_{k\in \{Dist(k,j)\leq d_{0}\}}P_{k}},$$

where *Dist*(*i, j*) is travel cost from *i* to *j*, *S_j_* is the supply capacity of service site *j*, *P_k_* is the number of people in population location *k*, respectively.

Step 2: Generate a catchment with *d*_0_ as threshold travel cost for each population location *i* and search all service sites within the catchment. Additionally, the spatial access index (SPAI) of *i*, $A_{i}^{F}$, is derived by summing the ratios of service sites within the catchment area of population location *i*:

(2) $$A_{i}^{F}=\sum_{j\in \{Dist(i,j)\leq d_{0}\}}R_{j}\;$$

The 2SFCA method has provided a widely accepted model to estimate healthcare accessibility since it was proposed [7,17,18,19]. Despite its popularity, the 2SFCA method has two major limitations. One limitation is that it assumes all locations within the given catchment area have equal access without a distance decay. In fact, people’s willingness to travel decreases with increasing distance. The other limitation of the 2SFCA method is that it utilizes fixed catchment size. For example, the catchment sizes of rural and urban areas should be different because people in rural areas are willing to travel further to seek medical services than those in urban areas. The subsequent research mainly aims at overcoming above two limitations.

### 2.2. Major Extensions of 2SFCA

Most existing pieces of literature extends the basic 2SFCA method from three aspects: the introduction of impedance function, the calculation of catchment sizes, and the competition effect of supply and demand.

#### 2.2.1. Extension on Introduction of the Impedance Function

It is generally accepted that the 2SFCA method is insufficient without the addition of impedance function [20,21,22,23]. Luo and Qi [22] presented an enhanced 2SFCA (E2SFCA) method by introducing Gaussian weights when calculating the supply-to-demand ratio. The Gaussian weights are calculated from the Gaussian function and utilized to reveal that people’s willingness to travel decreases with the increasing distance. This E2SFCA method works in two steps. First, a 30-min catchment around each service site *j* is generated and the catchment is divided into three subzones with a 10-min interval. The method of dividing the catchment area into several sub-areas is called the zonal method. Additionally, the supply-to-demand ratio *R_j_* is calculated by

(3) $$R_{j}=\frac{S_{j}}{\sum_{r=1,2,3}\sum_{k\in D_{r}}P_{k}W_{r}}\;$$

where *W_r_* is a Gaussian weight defined beforehand for the *r*th subzone *D_r_*. The spatial access index is then calculated by

(4) $$A_{i}^{F}=\sum_{r=1,2,3}\sum_{j\in D_{r}}R_{j}W_{r}\;$$

Some researchers have suggested that a sudden drop occurs at the edge of each zone when the zonal method is applied to large geographical areas [20,21]. Hence, some continuous impedance functions were developed. Mcgrail and Humphreys [23] proposed a continuous impedance function. In this function, a weight was assigned the value 1 for the first 10 min, the value 0 for more than 60 min, and a gradual decay for the time between 10 and 60 min. More continuous impedance functions, such as the Gaussian function [24,25], the kernel density function [20,26] and the gravity-based function (e.g., the power function, exponential function) [27,28,29] were subsequently introduced to model the distance decay effect. By generalizing the impedance function as a term *f*(*d*) [1], all measures in the 2SFCA method can be integrated as follows:

(5) $$A_{i}^{F}=\sum_{j\in \{Dist(i,j)\leq d_{0}\}}R_{j}f(d_{ij})=\sum_{j\in \{Dist(i,j)\leq d_{0}\}}\left(S_{j}f(d_{ij}\right)/\sum_{k\in \{Dist(k,j)\leq d_{0}\}}P_{k}f(d_{kj})),$$

where the impedance function, *f*(*d*), indicating how travel cost influences accessibility, can be a piecewise function or a continuous function.

The nature of this extension is to add an additional impedance function within the catchment area of 2SFCA. All references above are based on the extension of the impedance function and the difference between them mainly lies in the decay trend of impedance functions.

#### 2.2.2. The Extension on the Calculation of Catchment Sizes

The other limitation of the 2SFCA method is that it utilizes fixed catchment size. Luo and Whippo [30] proposed a new method named Variable 2SFCA(V2SFCA) to define the catchment sizes dynamically by increasing the catchment size progressively till a base population and a ratio of physician-to-population were met. This V2SFCA method can determine the appropriate catchment sizes effectively. However, the determination of the base population and the physician-to-population ratio is somewhat subjective, which is due to a lack of an adequate basis. McGrail and Humphreys [21] divided the catchment area into five levels based on population density to measure healthcare accessibility. Additionally, this extension is called Dynamic 2SFCA(D2SFCA). The D2SFCA has a strong practical significance, especially suitable for the case studies in areas where urban and rural areas are mixed. Tao et al. [27] demonstrated that different facilities may have different search radius and generally large-scaled facilities have large search radii. So different catchment sizes were assigned to each healthcare site according to its capacity. The results show that the multi-radius is superior to single search radius utilized in the basic 2SFCA when calculating accessibility. Jamtsho et al. [31] proposed a nearest-neighbor two-step floating catchment area (NN-2SFCA) model. This method assumes that the demand points only select a certain number of closest facilities within the search radius.

#### 2.2.3. The Extension Based on the Competition Effect of Supply and Demand

In addition to the above extensions for the two major limitations of 2SFCA, the competition effect between facilities is also considered by some scholars. Wan et al. [32] demonstrated that the potential competition between service sites would affect the population demand on sites and the population demand might be overestimated in the E2SFCA method. A three-step floating catchment area(3SFCA) method was proposed to minimize this overestimation. It assumed that people’s demand for a healthcare site is affected by the availability of other nearby sites. Specifically, a selection weight for each pair of demand-supply sites was introduced into this method, thus adjusting the population demand. This method is implemented in three steps. It first divides the catchment into 4 sub-zones (i.e., 10, 20, 30, and 60 min, respectively) and assigns a Gaussian weight to each service site based on the sub-zone in which it lies. Additionally, the selection weight *G_ij_* is computed by

(6) $$G_{ij}=\frac{T_{ij}}{\sum_{k\in \{Dist(i,k)\leq d_{0}\}}T_{ik}}\;$$

where *G_ij_* indicates the probability of selection on a service site, *T_ij_* and *T_ik_* are the predefined Gaussian weights for service site *j* and *k*, respectively. The second step is to calculate the adjusted supply-to-demand ratio *R_j_*:

(7) $$R_{j}=\frac{S_{j}}{\sum_{r=1,2,3,4}\sum_{k\in D_{r}}G_{kj}P_{k}W_{r}}\;$$

The third step is to calculate the SPAI of population location *i* by summing up the physician-to-population ratios of service site *j* within the catchment area of population location *i*:

(8) $$A_{i}^{F}=\sum_{r=1,2,3,4}\sum_{j\in D_{r}}G_{ij}R_{j}W_{r}\;$$

The 3SFCA method effectively minimizes the overestimation of the healthcare demand. However, the selection weight utilized in this method was just based on the travel time. In real life, people’s selection on a service site was not only related to travel time, but also affected by the capacity of the site. The Huff model is a gravity-based model proposed by Huff to survey the scale of retail stores [33]. The core idea of the Huff model is that consumers’ purchase probabilities are positively related to the attractiveness/capacity of stores and inversely related to its distance from consumers. So, the Huff model is commonly utilized to quantify the probability of people’s choice of a service site out of other nearby available ones. Luo [34] integrated the original Huff model and floating catchment area method to express population selection of services. The negative power distance impedance function is utilized in the original Huff model and the probability of the population location *i* in choosing service site *j*, *Prob_ij_*, is calculated by

(9) $$Prob_{ij}=\frac{S_{j}d_{ij}^{-\beta }}{\sum_{s\in D_{0}}S_{s}d_{is}^{-\beta }}\;$$

where *d_ij_* is the travel cost from *i* to *j*, *β* is a distance impedance coefficient indicating the extent of the distance decay, respectively. Luo [35] proposed an enhanced three-step floating catchment area (E3SFCA) method by modifying this negative power function with a Gaussian function. The modified Huff model is overwritten as follows:

(10) $$Prob_{ij}=\frac{S_{j}e^{-d_{ij}^{2}/\beta }}{\sum_{s\in D_{0}}S_{s}e^{-d_{is}^{2}/\beta }}\;$$

Instead of the predefined subzone-based Gaussian weight in previous studies, a continuous Gaussian weight is utilized to model the spatial interactions between the demand and supplies. Additionally, the adjusted supply-to-demand ratio *R_j_* and the SPAI of the population location *i* are computed respectively according to

(11) $$R_{j}=\frac{S_{j}}{\sum_{k\in D_{0}}Prob_{kj}P_{k}W_{kj}}\;$$

and

(12) $$A_{i}^{F}=\sum_{j\in D_{0}}Prob_{ij}R_{j}W_{ij}\;$$

The E3SFCA method can better adjust the population demand to overcome the overestimation and underestimation problems and, therefore, generate more reliable measures of spatial access to healthcare services.

## 3. An Improved 3SFCA Method

Despite many achievements in developing the 2SFCA method, problems remain. In previous studies, a common calculation method of travel time is to assign each road an empirical speed and then a conduct road network analysis [9,10,11], which ignores complicated traffic conditions in reality. Meanwhile, the individual healthcare demand of different age groups is considered equally, disregarding that the elderly usually have a higher healthcare demand than the young and thus the population demand is underestimated. To overcome the limitations of estimating travel cost and population demand, this paper improves the 3SFCA method. The improved method is based on more reasonable assumptions about the patient’s medical seeking behavior and medical needs for medical services.

When seeking medical care, especially in emergencies, patients are more likely to use taxis or private cars than buses or rail transit. An accurate estimation of driving time from the population location to hospitals is of crucial importance. In this paper, the driving time is precisely calculated by introducing real-time traffic conditions from AutoNavi. The AutoNavi distance measurement API is a travel distance calculation interface provided in the form of HTTP and the query data is returned in JSON or XML. Its strategy aims to avoid traffic jams, but it may take longer because of the possibility of a detour. People’s travel behavior of searching for medical care is highly consistent with this strategy. Given an origin (i.e., population location) and a destination (i.e., service site), the driving time between two points is achieved with the API. Thus, the AutoNavi API is utilized to estimate the driving time at different periods by programming in Asp.net.

**Figure 1:**
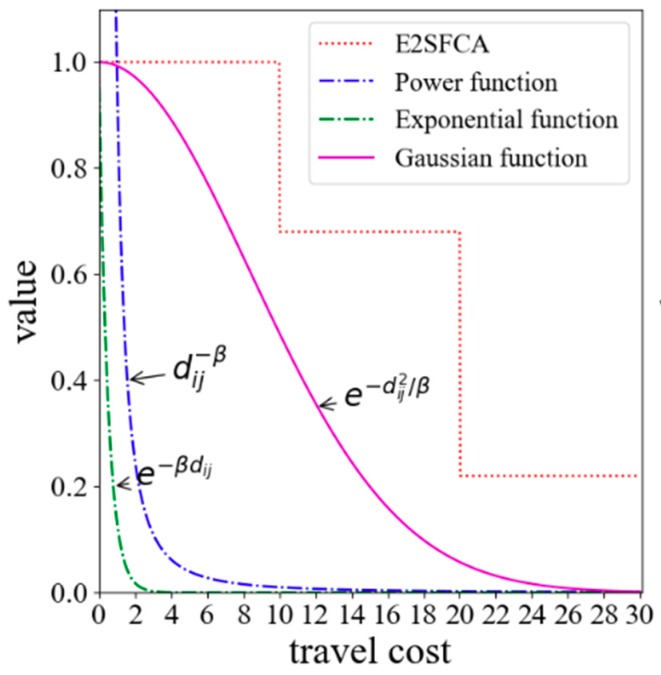
Major forms of impedance functions.

In general, people tend to go to neighboring hospitals that are rich in medical resources when seeking medical care. This health-seeking behavior is consistent with the core idea of the Huff model. So, the Huff model is utilized to calculate the probability of people seeking a service site. The key of the Huff model is the determination of the impedance function. So far, the most common forms of impedance functions are the negative power function ($f(d_{ij})=d_{ij}^{-\beta }$), the exponential function ($f(d_{ij})=e^{-\beta d_{ij}}$), and the Gaussian function ($f(d_{ij})=e^{-d_{ij}^{2}/\beta }$). As shown in Figure 1, the power function and the exponential function are convex functions. The convex function tends to decay too sharply near the origin when compared with the empirical evidence. However, the decay curve of the Gaussian function is S-shaped, and its decay rate increases first and then slows down with the increase of the distance. Since the Gaussian function relatively has a slow decline rate close to the origin, it is superior and can better reveal the effect of distance decay [36,37]. Therefore, the Gaussian function is utilized in this method as the impedance function. In general, 10 min is treated as an initial impedance with no decay [23]. Thus, the Gaussian function is revised as

(13) $$f(d_{ij})=\{\begin{array}{cc} 1 & d_{ij\leq 10}\\ e^{-{\left(d_{ij}-10\right)}^{2}/\beta } & 10<d_{ij}\leq d_{0}\\ 0 & d_{ij}>d_{0} \end{array}\;$$

where *d_ij_* is the travel time obtained in real-time traffic.

Both experience and statistics indicate that the elderly are at high risk of chronic diseases (e.g., heart disease, stroke, cancer, diabetes and lung disorders). Early literature has suggested that the prevalence of chronic diseases in the elderly is 2 to 3 times that of the general population [38,39]. According to the statistics, the prevalence ratio of chronic diseases among the aged is 53.8% and among which the urban aged is 77.7%. The prevalence ratio in cities is higher than that in rural areas. Moreover, the prevalence ratio varies according to the level of the city and the high-level cities have a high prevalence ratio [40]. In the latest research, a report entitled “China’s Pension Industry Development Strategy Research (2014)” released by China’s Center for Information Industry Development (CCID) Consulting pointed out that 80% to 90% of the aged are in the chronic and sub-healthy group and their demands for medical care are 3–5 times that of the young in China. Therefore, a demand weight index (DW) is introduced in this improved method to reveal the differences in the medical needs between the elderly and the young. The improved method is implemented in three steps:

Step 1: The probability of the population location *i* choosing service site *j*, *Prob_ij_*, is calculated by

(14) $$Prob_{ij}=\frac{C_{j}f(d_{ij})}{\sum_{s\in D_{0}}C_{s}f(d_{is})}\;$$

where *f*(*d_ij_*) is the revised Gaussian function (see Equation (13)).

Step 2: We assume that the medical needs of the elderly are DW times as big as the young. Thus, the adjusted population demand in location *k*, *P_k_*, is revised as follows:

(15) $$\begin{array}{ll} P_{k} & =DW*Pop_{oldk}+Pop_{elsek}\\ & =DW*Pop_{oldk}+Pop_{allk}-Pop_{oldk}\\ & =Pop_{allk}+(DW-1)*Pop_{oldk} \end{array}$$

where $Pop_{allk}$ is the total number of people in the population location *k*, $Pop_{oldk}$ is the count of the elderly in the population location *k*, *DW* is a demand weight index revealing the differences in the medical needs between the elderly and the young. With the adjusted population demand *P_k_*, the supply-to-demand *R_j_* is implemented by

(16) $$R_{j}=\frac{S_{j}}{\sum_{k\in D_{0}}Prob_{kj}P_{k}f(d_{kj})}\;$$

Step 3: Compute the spatial access index of population site *i* by

(17) $$A_{i}^{F}=\sum_{j\in D_{0}}Prob_{ij}R_{j}f(d_{ij})\;$$

This improved method considers real-time traffic when calculating travel time, and thus dynamic results of accessibility are derived, which is conducive to a more comprehensive understanding of the accessibility in different time periods. Besides, it assumes that the elderly have a higher demand for medical resources than the young, which is a logical assumption in accordance with reality. Hence, the population demand has increased compared with previous methods assuming that the medical needs of the elderly and the young are the same.

## 4. A Case Study

### 4.1. Study Area and Data

**Figure 2:**
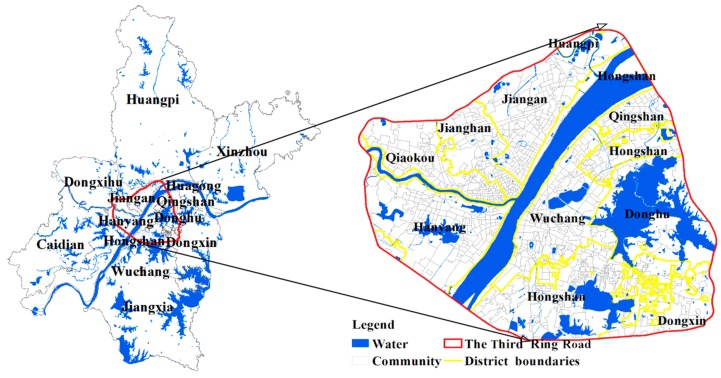
The study area.

Wuhan is the provincial capital of Hubei in central China. It has abundant water resources. This city has 13 municipal districts with a total area of 8494.41 square kilometers and has 10,914 thousand permanent residents in 2017. As shown in Figure 2, the study area is the main urban area of Wuhan as well as the core functional area.

**Figure 3:**
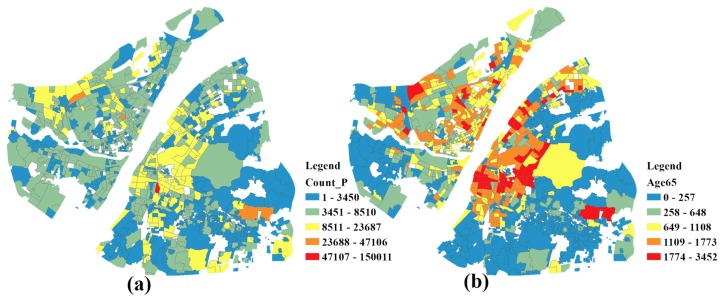
(**a**) The population distribution map; (**b**) the elderly distribution map.

The study area mainly covers 7 districts (i.e., the Jiang’an, Jianghan, Qiaokou, Hanyang, Wuchang, Hongshan and Qingshan Districts), which consists of 926 communities. Figure 3a,b show the distribution of the population and the elderly, respectively. The elderly mainly inhabit the Wuchang and Jianghan Districts.

**Figure 4:**
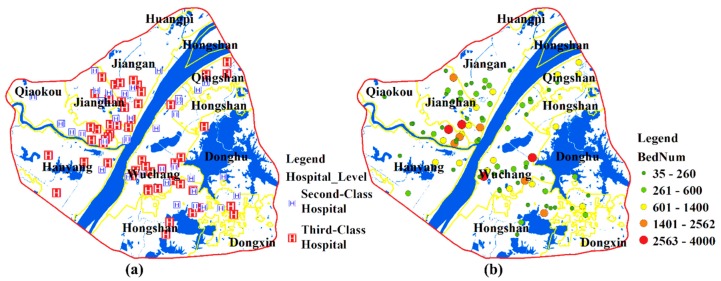
(**a**) A distribution map of the hospitals; (**b**) the distribution of the number of beds.

Hospitals are divided into 3 levels (first-, second- and third-class hospitals) in the Chinese healthcare system. The third-class hospitals are superior while the first-class hospitals are inferior in terms of function, facilities, and technical strength, and the second-class hospitals are in between. Some researchers have indicated that the patients’ willingness to travel is closely related to the healthcare provider [41,42]. In general, patients tend to choose hospitals that are professional and have a good reputation (e.g., the second- or third-class hospitals in China) [43,44,45]. This study focuses on the second- and third-class hospitals which represent the major medical resources of the city. A total of 88 hospitals are included in this paper. The number of beds and health technicians are obtained from the corresponding official websites of the hospitals. The distribution of hospitals and the number of beds in each hospital are shown in Figure 4a,b. Most medical resources are concentrated in the Wuchang and Jianghan Districts.

### 4.2. Data Preprocessing

#### 4.2.1. Estimation of Population Locations

**Figure 5:**
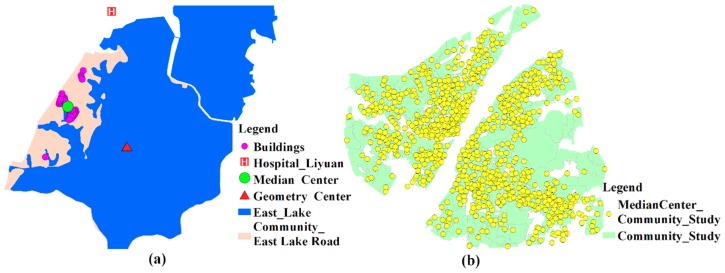
(**a**) The geometry center and median center of East Lake Road Community; (**b**) median centers of the study communities.

Previous studies usually utilize the geometry center of the research unit to represent population location when calculating the travel time to healthcare services. However, this method is not applicable in communities with imbalanced population distributions. Many communities in Wuhan contain some areas of water, where few people reside. As shown in Figure 5a, the community, named East Lake Road Community, is largely occupied by water and people here basically live in the western part of the community. As a result, the geometry center and population location are obviously inconsistent. However, the median center is a central trend measure and its total Euclidean distance to all elements in the data set is minimal. Therefore, the median centers of buildings are regarded as the population location of each community in this paper. The population locations of communities in Wuhan is illustrated in Figure 5b.

#### 4.2.2. Estimation of Hospital’s Beds

The number of beds and health technicians is obtained from the corresponding official websites of hospitals. For some reasons, there may be data missing. Data missing generally occurs in the following 2 scenarios and the corresponding solutions are as follows:

Scenario 1: The number of beds is missing while the number of health technicians is available;

Solution 1: Calculate the missing data according to the standard for the classification of hospitals shown in Table 1. For example, if the number of health technicians of a third-class hospital is 550 while the number of beds is missing, the number of beds can be calculated by 550/1.03 with a result of 534.

Scenario 2: The number of beds and the number of health technicians are both missing.

Solution 2: Mean completer method is a widely used imputation method [46]. The number of beds is numerical, so the missing values are filled with the average number of beds in other hospitals of that class. For example, given that a hospital is a second-class hospital and the data of the number of beds and health technicians are both missing if the average number of beds in other second-class hospitals is 350, the number of beds in this hospital can be assigned the value 350.

### 4.3. Evaluation Procedure

The evaluation procedure is composed of three steps. Firstly, the sensitivity of SPAI is assessed for the improved method. Secondly, healthcare accessibility results during different time periods are implemented. Thirdly, the crowdedness of hospitals is calculated and hospital beds shortage areas are identified.

As mentioned in Section 3, the demand for medical care among the elderly is 3–5 times that of the young in China and the high-level cities have a high prevalence ratio. As one of the biggest cities in China, the DW of Wuhan is set to 5 in this paper.

#### 4.3.1. Sensitive Assessment

No matter the 2SFCA method or its developments, the choice of the impedance coefficient is arbitrary. Since the impedance coefficient reflects the extent to which people’s willingness to travel decays over time, it should be determined based on actual surveys of healthcare utilization behavior. However, this takes a lot of manpower and capital investment, and the findings of specific case areas may not be extended to other areas, so they are not highly operational. Thus, with the lack of a sufficient basis for parameter setting of the impedance function, more comprehensive results should be provided as references through multi-scenario analysis, and sensitivity assessment should be conducted for the setting of the key parameter *β*.

**Figure 6:**
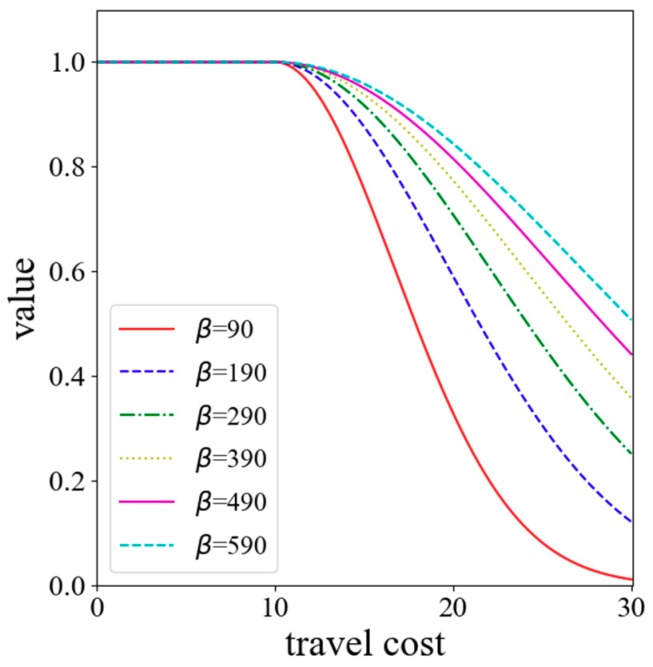
The revised Gaussian function with different impedance coefficients (*β*).

Thirty minutes has been considered an appropriate catchment size to analyze the healthcare accessibility [15,47]. Kwan [48] suggested that value 0.01 is a critical value for the Gaussian function approaching 0. As shown in Figure 6, for travel cost (i.e., 30 min), the *β* value of 90 corresponds to the Gaussian value of 0.01. Therefore, the minimum value of *β* is set to 90 so that the Gaussian value is always greater than 0.01. The maximum value is set to 590 because the curve is relatively ‘flat’ at this point [49]. Six impedance coefficients, which range from 90 to 590 with an increment of 100, are used to evaluate the stability of SPAI.

#### 4.3.2. Healthcare Accessibility Analysis During Different Time Periods

As indicated in Section 3, it is inaccurate to evaluate travel time without considering an actual traffic situation. Comparing the accessibility during different time periods (e.g., at rush hours or at non-peak periods) helps to understand the accessibility more comprehensively as the traffic situation changes, and thus providing a basis for policy-makers to make relevant policies.

Road congestion varies during different time periods. Generally, the morning peak is from 7:00 a.m. to 9:00 a.m. and the evening peak is from 5:00 p.m. to 7:00 p.m. During rush hours, some major roads are regularly choked with traffic and the travel cost will increase dramatically. Thus, based on the traffic condition, the consulting hours of hospitals (i.e., generally from 7:00 a.m. to 6:00 p.m.) are divided into 5 time periods. These 5 time periods are 7:00 a.m. to 9:00 a.m., 9:00 a.m. to 12:00 p.m., 12:00 p.m. to 2:30 p.m., 2:30 p.m. to 5:00 p.m., and 5:00 p.m. to 6:00 p.m. respectively. By dividing consulting hours, a dynamic accessibility result is obtained. Specifically, this study mainly obtained the travel time from community to healthcare site at following 5 time points: 07:40 a.m., 10:40 a.m., 12:40 p.m., 3:20 p.m., and 5: 20 p.m. Besides, in consideration of the extent to which travel time affects people’s travel choices, a strong distance impedance is utilized in this study, namely, *β* is set to 90.

#### 4.3.3. Calculating the Crowdedness of Hospitals and Identifying Shortage Areas

An important goal of calculating spatial accessibility is to identify shortage areas of healthcare services and then take measures to minimize inequities. In the latest study, an inverted two-step floating catchment area (i2SFCA) method is introduced to capture the “crowdedness” (scarcity of resources or intensity of competition) for facilities [50]. The i2SFCA method is an extension of the classic Huff model. The crowdedness is a ratio of population served to supply capacity (e.g., patients per sickbed). Specifically, it is calculated as

(18) $$C_{j}=V_{j}/S_{j}=\sum_{i=1}^{m}\left[D_{i}f(d_{ij})/\sum_{l=1}^{n}\left(S_{l}f(d_{il})\right)\right]\;$$

where *C_j_* is the demand-to-supply ratio at service site *j*, *V_j_* is the total number of population attracted by site *j*, *S_j_* is the capacity of site *j*, *D_i_* is the population size at population location *i*, *m* and *n* are the number of population locations and service sites, respectively, and *f*(*d*) is a generalized impedance function and its specific form utilized in this paper is the Gaussian function. A high crowdedness value means that the facility is crowded with patients. The crowdedness of hospitals in the study area is calculated based on the i2SFCA method.

According to the Norm for the Urban Public Facilities Planning (GB50442-2008), which is utilized as a guide to make urban public facilities planning more scientific, the city scale and the number of beds per thousand people are determined. Table 2 shows the relationship between population size and city scale. Table 3 is the standard of number of beds per thousand people. Since the population size of Wuhan is larger than 2 million, the number of beds per thousand people should be larger than 7. In this paper, the number of beds is used as the capacity of hospitals, and thus the actual indicator of accessibility is the number of beds available per people. Thus, 0.007 is a reasonable threshold value to identify areas where medical resources are scarce.

## 5. Results

### 5.1. Results of Sensitivity Assessment

**Figure 7:**
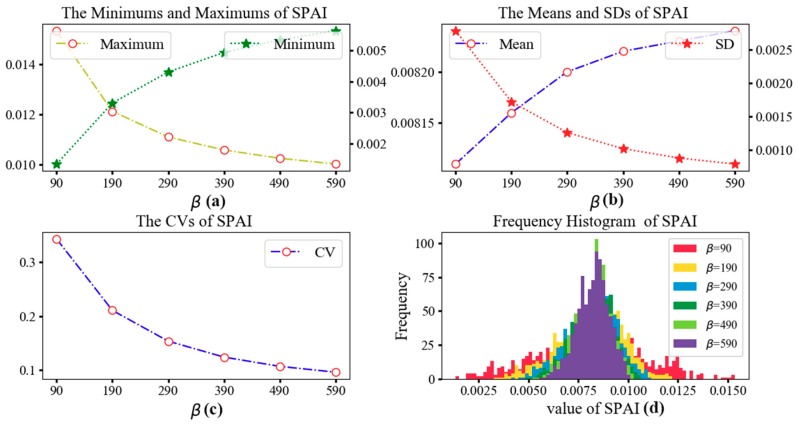
(**a**) The minimums and maximums of SPAI; (**b**) The means and SDs of SPAI; (**c**) The CVs of SPAI; (**d**) The frequency histogram of SPAI.

Table 4 shows the minimum, maximum, mean, standard deviation (SD), and coefficient of variation (CV) values of SPAI with different distance impedance coefficients *β*. CV is a statistic to measure the variation degree of each observation and calculated by the ratio of SD to mean. As shown in Table 4, the mean value remains almost unchanged while the CVs decrease sharply with the increase of *β*. Since the increase of *β* indicates the weakening of distance impedance effect, the decrease of CVs implies that a weaker distance impedance results in a lesser extent of the variance of spatial accessibility. The statistical values are further illustrated in Figure 7. With the increasing of *β*, the values of SPAI become more concentrated. In other words, the difference in spatial accessibility between communities is narrowed.

**Figure 8:**
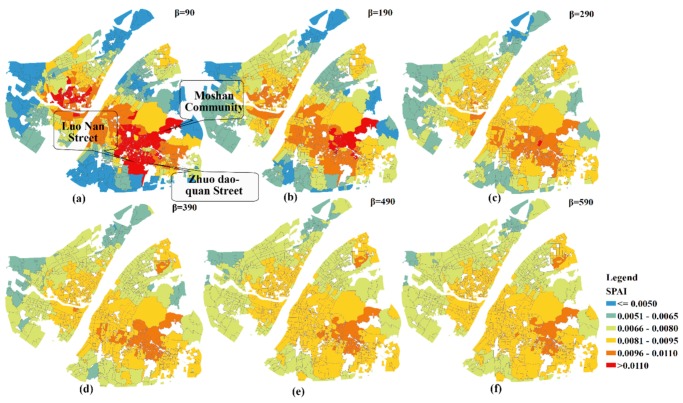
The geographic patterns of SPAI with different *β* values. (**a**) *β* = 90; (**b**) *β* = 190; (**c**) *β* = 290; (**d**) *β* = 390; (**e**) *β* = 490; (**f**) *β* = 590.

The geographic patterns of SPAI for different extents of distance impedance coefficients are shown in Figure 8. For the purpose of comparison, the values of SPAI are categorized into the same intervals (i.e., <=0.005, 0.0051–0.0065, 0.0066–0.0080, 0.0081–0.0095, 0.0096–0.0110, and >0.011). It can be observed that the geographic patterns of SPAI change significantly among the first three impedance coefficients. For example, the SPAI values of almost all communities in Luonan Street and Zhuodaoquan Street are greater than 0.011 when *β* is 90. However, only one community has large SPAIs (e.g., SPAI > 0.011) when *β* is 290. Besides, the geographic patterns of SPAI remain unchanged when *β* is bigger than 290. It can be concluded that a faster decay speed will lead to a greater difference in accessibility between communities. Therefore, the SPAI values of hospital adjacent areas are significantly higher than that of other regions which are away from the hospital.

### 5.2. Dynamic Healthcare Accessibility Analysis

**Figure 9:**
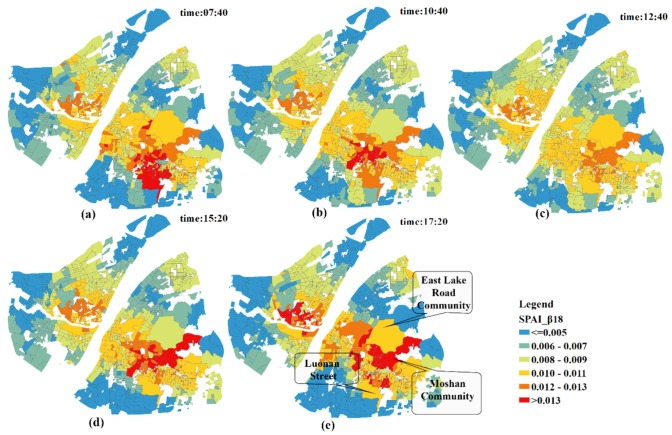
The geographic patterns of SPAI at different points-in-time. (**a**) 07:40; (**b**) 10:40; (**c**) 12:40; (**d**) 15:20; (**e**) 17:20.

The geographic patterns of SPAI during different time periods are shown in Figure 9. During the rush hours (e.g., 07:40 and 17:20), the number of communities characterized by a large SPAIs is significantly higher than that of other times in this study. Additionally, during the flat hump period (e.g., at 12:40), almost all communities have no large SPAIs and the difference in accessibility between communities is narrowed.

**Figure 10:**
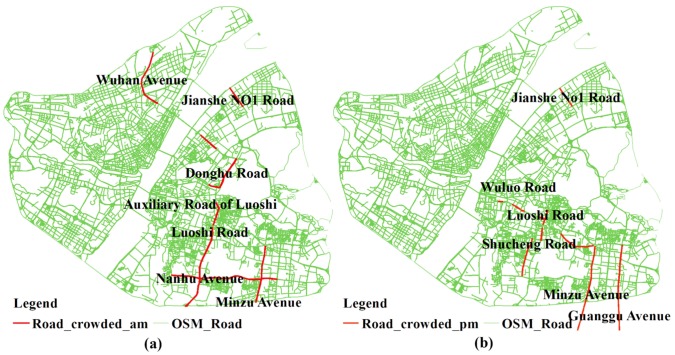
The congestion road sections in rush hour. (**a**) morning peak; (**b**) evening peak.

As for the local analysis, the SPAIs of communities around the East Lake Road Community, Moshan Community and Luonan Street present the biggest change. When coupled with the information in Table 5 and Figure 10, it can be concluded that traffic congestion has significantly increased the accessibility of the communities near hospitals, and the difference in spatial accessibility to healthcare services between communities is enlarged. That is, worse traffic conditions lead to a greater distance impedance effect between hospitals and the communities far from hospitals. As a result, the competition between communities near hospitals and those relatively far away from hospitals is decreasing, and the spatial accessibility of communities near hospitals which are less affected by traffic is dramatically increasing.

### 5.3. Results of Hospitals Crowdedness and Identifying Shortage Areas

**Figure 11:**
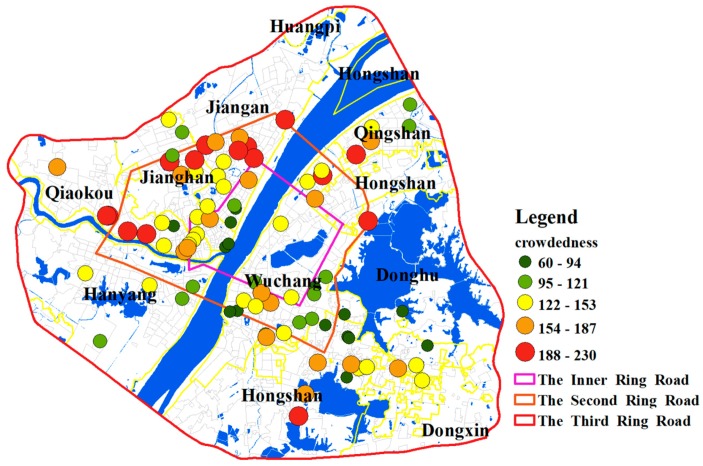
The crowdedness of hospitals.

The crowdedness of hospitals is shown in Figure 11. The hospitals within the Inner Ring Road or outside the Second Ring Road are less crowded while those between them are relatively more crowded. Besides, the hospitals in the Jiang’an District generally have higher crowdedness values. The high value of crowdedness indicates that hospitals are in short supply and the number of beds in these hospitals needs to increase. Additionally, the hospitals at the boundary of the Third Ring Road are less crowded. However, since the population demand of areas outside the boundary is not considered, the crowdedness values of hospitals at the boundary might be inaccurate.

**Figure 12:**
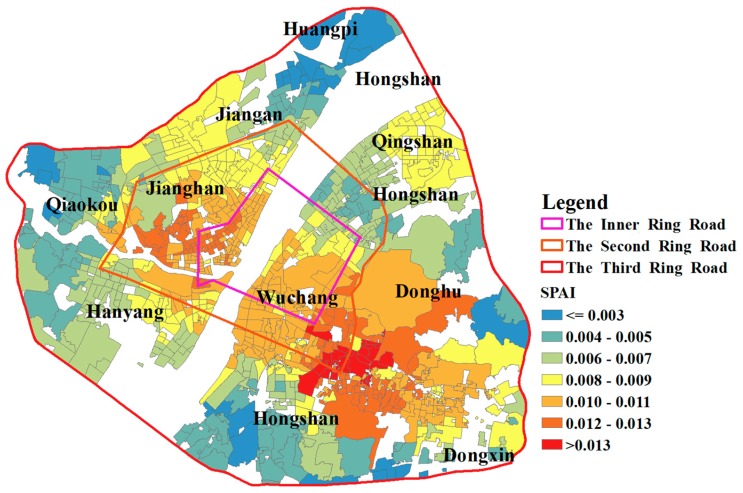
The geographic pattern of SPAI.

A concentric geographic pattern of SPAI is shown in Figure 12. The healthcare accessibility declines as the distance to the core of the study area increases. As indicated in Section 4.3.3, the shortage areas are determined as areas whose spatial access index is smaller than 0.007. There are 330 communities that are identified as shortage areas. The demand for beds in most communities of the study area is met. Additionally, most of these shortage areas are in the suburbs, which implies that the medical infrastructure in suburban areas needs to be strengthened. The SPAIs near the boundary in the study area are not referenced because hospitals outside the boundary are not considered, which is likely to affect the calculation of supply in the catchment of population locations.

## 6. Discussion and Conclusions

In this paper, two important variables (travel cost and population demand) of measuring healthcare accessibility are improved. Specifically, real-time traffic information is introduced to calculate the travel time and a demand weight index is used to adjust the population demand.

By changing the distance impedance coefficient *β*, the sensitivity assessment of accessibility is carried out in this study. The comparison result shows that a strong distance impedance will enlarge the accessibility difference between communities. Besides, traffic congestion has significantly increased the accessibility of communities near hospitals by keeping distant communities from accessing healthcare services. As a result, the difference in spatial accessibility between communities is enlarged. Based on the i2SFCA method, the crowdedness of hospitals in the study area is calculated. The results show that hospitals within the Inner Ring Road or outside the Second Ring Road of Wuhan are less crowded while those between them are more crowded. Notably, hospitals in the Jiang’an District generally have a higher crowdedness, which implies that hospitals in this area are in short supply and the number of beds in these hospitals needs to be increased. Finally, 0.007 is used as a threshold value for designating hospital beds shortage areas and the result shows that most of these shortage areas are in the suburbs, which implies that the medical infrastructure in suburban areas needs to be strengthened.

The major advantage of this improved method compared to previous models lies in its reasonable assumptions about patient’s medical seeking behavior and medical needs for medical services. With the utilization of real-time traffic, the travel time is accurately estimated. Additionally, dynamic results of healthcare accessibility are derived during different time periods, which helps to understand the accessibility more comprehensively. Using a demand weight index, the differences between the elderly and the young in the needs of medical facilities are clearly reflected and one can, therefore, better adjust the population demand.

Despite the notable advantage, several issues deserve particular attention when implementing this method in healthcare accessibility studies. First, the determination of the impedance coefficient *β* is somewhat arbitrary. As suggested by Huff and McCallum [51], it should be determined based on actual surveys of healthcare utilization behavior. Future researches may focus on exploring the health-seeking behavior of patients by utilizing taxi GPS trajectory data or pedestrian-based trajectory data collected by mobile phones, which will help to calibrate the impedance coefficient. Second, a fixed 30-min catchment size is utilized since the study area of this paper is the main urban area of Wuhan city, which is a relatively small area. However, if the study area is expanded to an entire city or a larger area, a variable or a dynamic catchment size is required. Third, the utilization of the AutoNavi API plays an important role when calculating travel time. Although AutoNavi proves to be a very powerful tool in the field of route planning, its calculation process remains a black box and cannot be controlled by the researchers.

In conclusion, this improved method provides a new perspective for analyzing healthcare accessibility and shows great potential in the allocation works of healthcare resources.

## Figures and Tables

**Table 1:** The standard for classification of hospitals.

Level	Number of Beds	Technician Number
first-class	≤100	0.7 * Number of beds
second-class	100–500	0.88 * Number of beds
third-class	>500	1.03 * Number of beds

* refers to a multiplication sign.

**Table 2:** The city scale standard.

City Scale	Small City	Middle City	Large City		
			I	II	III
Population Size (ten thousand)	<20	20–50	50–100	100–200	≥200

**Table 3:** The standard of the number of beds per thousand people.

City Size	Small City	Middle City	Large City		
			I	II	III
Number of beds per thousand people	4–5	4–5	4–6	6–7	≥7

**Table 4:** The descriptive statistics of the results.

Distance Impedance Coefficient (*β*)	Minimum	Maximum	Mean	SD	CV
90	0.00134	0.01534	0.00811	0.00278	0.34245
190	0.00331	0.01213	0.00816	0.00172	0.21049
290	0.00431	0.01111	0.00820	0.00126	0.15365
390	0.00494	0.01059	0.00822	0.00102	0.12416
490	0.00535	0.01026	0.00823	0.00088	0.10705
590	0.00564	0.01003	0.00824	0.00079	0.09632

**Table 5:** The top ten congestion road sections in rush hour provided by the Wuhan Traffic Management Bureau.

Rank	Road Name (a.m.)	Road Name (p.m.)
1	Donghu Road	Luoshi Road
2	Luoshi Road	Minzu Avenue
3	Tangxunhu North Road	Auxiliary Road of Wuluo
4	Daxueyuan Road	Daxueyuan Road
5	Auxiliary Road of Donghu	Shucheng Road
6	Wuhan Avenue	Wuluo Road
7	Minzu Avenue	Huquan Street
8	Nanhu Avenue	Guanggu Avenue
9	Jianshe No1 Road	Julong Avenue
10	Auxiliary Road of Luoshi	Jianshe No1 Road
